# Oncological aspects related to non-surgical treatment of basal cell carcinoma. Comments on: chemotherapeutical treatment of basal cell carcinoma with bleomycin via microinfusion of the drug into the skin (MMP®)^☆^

**DOI:** 10.1016/j.abd.2023.08.007

**Published:** 2023-12-08

**Authors:** Ivanka Miranda de Castro Martins, Ana Cláudia Cavalcante Espósito, Hélio Amante Miot

**Affiliations:** a Department of Infectology, Dermatology, Imaging Diagnosis and Radiotherapy, Faculty of Medicine, Universidade Estadual Paulista, Botucatu, SP, Brazil; b Service of Dermatology, Universidade do Oeste Paulista, Presidente Prudente, SP, Brazil

*Dear Editor,*

As the incidence of basal cell carcinoma (BCC) is increasing, investigations into treatments that are less costly and complex than surgery are invaluable.^1^ Therefore, we read with interest the article by Pacola et al., who performed the administration of intratumoral bleomycin, using a tattoo machine, in a series of 98 lesions followed for six months, resulting in a histopathological cure rate of 96.9% (95% CI 92.9%‒99.0%). And we would like to comment on certain oncological aspects regarding the generalization of the results.^2^

Intratumoral bleomycin, in association with electrochemotherapy, and/or intravenous bleomycin were reported to be effective in the treatment of BCC in 32 previous studies, achieving cure rates of 92% after two months of follow-up, with recurrences not precluding retreatment.^3^ This may be invaluable in patients without clinical conditions for surgery; however, the lack of randomized controlled trials comparing it with excisional surgery does not allow critical insight into the cost-effectiveness of chemotherapy infusion in the treatment of common BCC.

Due to the low mitotic rate, the assessment of histopathological recurrence is an adequate outcome for investigating the efficacy of therapies in BCC. However, the recurrence rate increases with the years of follow-up, and only 32% of them are detected in the first year following surgery,^4^ which makes the evaluation at six months insufficient for a satisfactory comparison with the oncological literature. Recurrence may be clinically imperceptible for many months, meaning that punch biopsy sampling, as used by Pacola et al., may underestimate its occurrence in another topography of the lesion. Especially because the scarring aspect promoted by bleomycin can make clinical and/or dermoscopic evaluation of recurrence difficult. Ideally, a follow-up of more than three years and histopathological sampling of the entire scar area is necessary for a correct estimate of the cure rate.

Additionally, factors associated with BCC recurrence are very diverse, including size, topography, margin size, histopathological type, and immunosuppression. The authors included high-risk lesions in the case series, applied different safety margin sizes, and treated lesions greater than 3 mm in depth, which are beyond the reaching scope of the tattoo machine needles. Furthermore, BCC lesion sampling by biopsy does not allow an accurate estimation of the depth and infiltrative components of the BCC as a whole.^5^

Finally, it is worth mentioning the high rate (19%) of loss to follow-up of patients included in the case series, without clarification whether it was due to treatment, adverse effects, or recurrence of lesions.

## Financial support

None declared.

## Authors’ contributions

Ivanka Miranda de Castro Martins: Design of the study, drafting and editing of the manuscript and approval of the final version of the manuscript.

Ana Cláudia Cavalcante Espósito: Design of the study, drafting and editing of the manuscript and approval of the final version of the manuscript.

Hélio Amante Miot: Design of the study, drafting and editing of the manuscript and approval of the final version of the manuscript.

## Conflicts of interest

None declared.
